# Spontaneous Upper Urinary Tract Rupture Caused by Ureteric Stones: Clinical Characteristics and Validation of a Radiological Classification System

**DOI:** 10.3390/diagnostics11091568

**Published:** 2021-08-29

**Authors:** Carolina Bebi, Matteo Giulio Spinelli, Gianpaolo Lucignani, Pierpaolo Biondetti, Laura Martinetti, Irene Fulgheri, Elisa De Lorenzis, Giancarlo Albo, Annamaria Ierardi, Franco Palmisano, Andrea Salonia, Gianpaolo Carrafiello, Emanuele Montanari, Luca Boeri

**Affiliations:** 1 Department of Urology, Foundation IRCCS Ca’ Granda–Ospedale Maggiore Policlinico, University of Milan, 20122 Milan, Italy; carolina_bebi@hotmail.it (C.B.); matteogiuliospinelli@gmail.com (M.G.S.); gplucignani@gmail.com (G.L.); elisa.delorenzis@gmail.com (E.D.L.); albo.giancarlo@gmail.com (G.A.); franco.palmisano@hotmail.it (F.P.); emanuele.montanari@unimi.it (E.M.); 2 Department of Radiology, Foundation IRCCS Ca’ Granda–Ospedale Maggiore Policlinico, University of Milan, 20122 Milan, Italy; pierpaolo.biondetti@gmail.com (P.B.); laura.martinetti@policlinico.mi.it (L.M.); irene.fulgheri@gmail.com (I.F.); annamaria.ierardi@policlinico.mi.it (A.I.); gianpaolo.carrafiello@policlinico.mi.it (G.C.); 3 Department of Clinical Sciences and Community Health, University of Milan, 20122 Milan, Italy; 4 Division of Experimental Oncology, Unit of Urology, URI, IRCCS Ospedale San Raffaele, 20131 Milan, Italy; salonia.andrea@hsr.it

**Keywords:** spontaneous upper urinary tract rupture, ureteric stones, radiology, classification system, emergency

## Abstract

Background: This study seeks to validate a radiological classification system of spontaneous upper urinary tract rupture (sUUTR) and to analyse its relationship with clinical, laboratory and radiological characteristics of sUUTR. Methods: We analysed data from 66 patients with a computerised tomography (CT)-proven sUUTR treated with ureteral or nephrostomy catheter positioning. Comorbidities were scored with the Charlson Comorbidity Index (CCI). All CT scans were reviewed by two experienced radiologists and one urologist, who classified sUUTR in (a) local spread, (b) free fluid and (c) urinoma. Interobserver agreement for radiological score was evaluated with the Intraclass Correlation Coefficient (ICC) and Cohen’s Kappa analyses. Descriptive statistics and logistic regression models verified the association between clinical variables and sUUTR severity. Results: The interobserver agreement for sUUTR classification was high among radiologists and between the radiologists and the urologist (all Kappa > 0.7), with an overall high interrater reliability (ICC 0.82). Local spread, free fluid and urinoma were found in 24 (36.4%), 39 (59.1%) and 3 (4.5%) cases, respectively. Patients with free fluid/urinoma had higher rate of CCI ≥ 1 than those with local spread (40.5% vs. 16.7%, *p* = 0.04). Intraoperative absence of urine extravasation was more frequently found in patients with local spread than those with free fluid/urinoma (66.7% vs. 28.6%, *p* < 0.01). Multivariable logistic regression analysis revealed that local spread (OR 4.5, *p* < 0.01) was associated with absence of contrast medium extravasation during pyelography, after accounting for stone size, fever and CCI. Conclusions: The analysed sUUTR classification score had good inter/intra-reader reliability among radiologists and urologists. Absence of urine extravasation was five times more frequent in patients with local spread, making conservative treatment feasible in these cases.

## 1. Introduction

Spontaneous Upper Urinary Tract Rupture (sUUTR) is a urine leakage that is not caused by external trauma or compression, destructive kidney lesions, recent urologic instrumentation or other iatrogenic causes [1]. It can occur at any level of the upper excretory system, from the renal calices to the ureterovesical junction, although it most frequently affects renal fornices [2].

sUUTR is a rare entity, reported in the literature either by urologists in the form of single case reports [3] and small-scale chart or literature reviews [2,4,5,6], or by radiologists as descriptive articles and perspectives [7,8].

The pathogenesis underlying excretory system disruption is believed to be an increase in intraluminal pressure due to obstruction, most commonly caused by ureteral stones [2,4,6] and less frequently by extrinsic mechanical compression of the urinary tract due to disparate etiologies such as pregnancy [9], gynaecological malignancies [10], metastatic gastric cancer [11] or abdominal aortic aneurism [12], among others. Moreover, when rupture occurs, its causative agent may sometimes remain undetected [3,13].

When a calculus is identified, symptoms at presentation may range from a renal colic [11,12,13,14] to those of an acute abdomen [15,16]. Notably, clinical and laboratory parameters may not be in line with the severity of urine extravasation, which is diagnosed radiologically with Contrast Enhanced Computed Tomography (CECT) scans. A recent study has proposed a new CECT-based classification of sUUTR into three categories: (i) Local Spread: fluid collection in the perinephric area; (ii) Free Fluid (urinary ascites): large fluid collection in perinephric area extending along the ureter in the retroperitoneum; and (iii) Urinoma: encapsulated fluid collection surrounded by a fibrous capsule [6]. Authors retrospectively reviewed 31 sUUTR cases and showed that most of them were located in a calyceal fornix and were caused by small distal ureteral stones [6]. However, validation of this sUUTR classification system is lacking [6].

Our study aims to perform a solid validation of the recent sUUTR classification system [6] by testing interrater agreement between two radiologists and one urologist and to investigate clinical, laboratory and radiological characteristics according to sUUTR severity.

## 2. Materials and Methods

We performed a retrospective chart review of patients consecutively presenting to the Emergency Department (ED) of IRCCS Foundation Ca’ Granda–Maggiore Policlinico Hospital from September 2014 to October 2020 and undergoing urological evaluation (any reason). By analysing the ED records, patients were screened according to the diagnosis at discharge, and for the specific purpose of this study, we included only patients who had a computerised tomography (CT)-proven sUUTR sustained by ureteral stones.

Clinical presentation and patients’ demographics including age and comorbidities were recorded. Comorbidities were scored with the Charlson Comorbidity Index (CCI) [17]. For the specific purpose of the analysis, CCI was categorised as 0 or ≥1. Body mass index (BMI), defined as weight in kilograms by height in square meters, was calculated for each patient. Max body temperature was recorded at ED admission. Complete blood count, platelet count, electrolytes, C-reactive protein, liver enzymes, serum protein, serum bilirubin and serum creatinine were measured in all patients. Stone diameter, location and degree of hydronephrosis were collected.

According to our internal protocol, patients with symptoms suggestive for ureteral stones underwent a whole-abdomen ultrasound (US) as first imaging modality. Cases with possible sUUTR (e.g., perirenal fluid collection) at US were submitted to CT with delayed images obtained 5–20 min after contrast medium injection.

All patients with sUUTR were treated with ureteral catheter or nephrostomy tube placement based on surgeon’s preference or patient factors. Parenteral broad-spectrum antibiotic prophylaxis was administered in all patients if not started in the ED before surgery. During surgery, a renal pyelography was performed to clearly identify the anatomy of the collecting system and to confirm urine extravasation.

### 2.1. Imaging Technique

A dual source, dual energy CT scanner (Siemens Somatom Definition Flash) was used for all cases. Unprocessed data acquired on axial plane with a slice thickness of 0.6 mm or 1.2 mm were processed and 3 mm slice axial images were obtained from the non-contrast and delayed CT phase.

One experienced radiologist in urological emergency (Radiologist 1), one junior radiologist (Radiologist 2) and one consultant urologist, blinded to each other, retrospectively reviewed all CT images with picture archiving and communication system (PACS) software in the absence of any information regarding the clinical and laboratory findings of the patients. 

Hydronephrosis was categorised according to the classification proposed by the Society for Fetal Urology [18]. 

**Figure 1:**
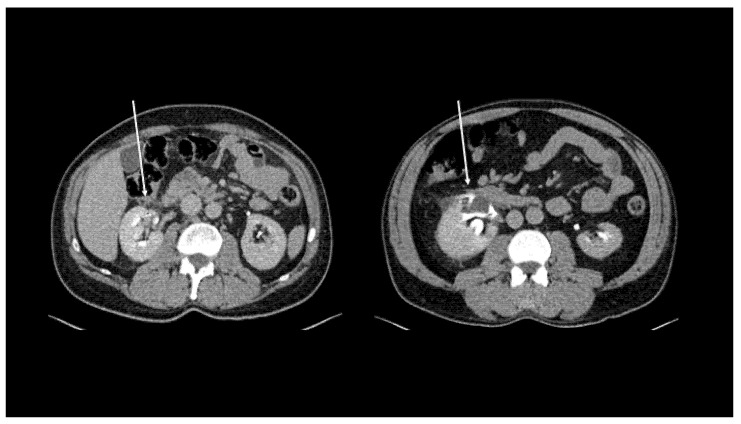
Axial computerised tomography characteristics of local spread at delayed excretory phase. Arrows indicate the leakage point.

**Figure 2:**
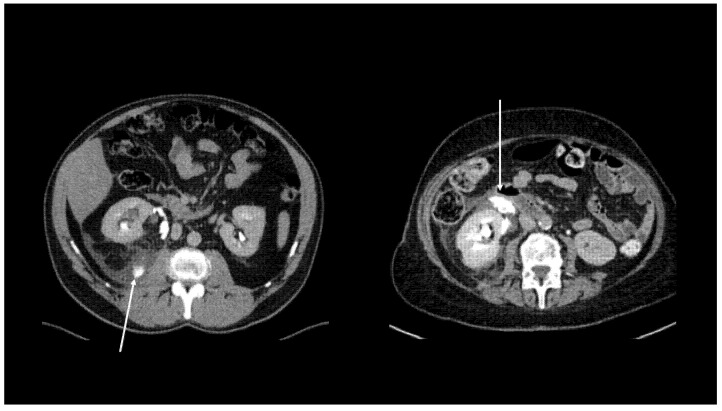
Axial computerised tomography characteristics of free fluid at delayed excretory phase. Arrows indicate contrast medium extravasation in the retroperitoneum.

**Figure 3:**
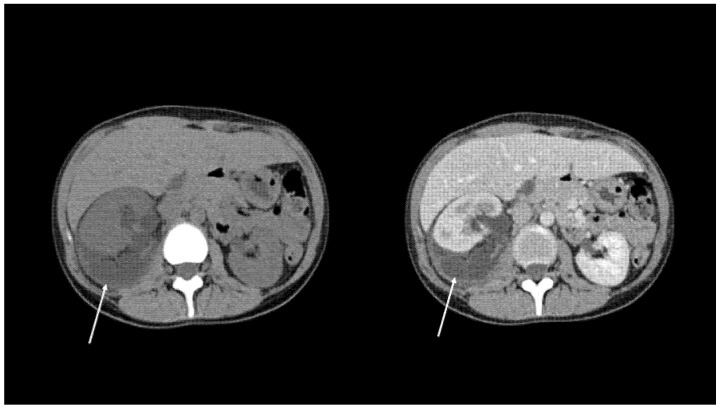
Axial computerised tomography characteristics of urinoma at non contrast CT and delayed excretory phase. Arrows indicate encapsulated fluid collection surrounded by a fibrous capsule.

The site of the leakage and the extent of the extravasation in the coronal and sagittal 3D reformatted images were recorded and the leakage was categorised as previously reported [6]: (i) Local Spread: fluid collection in the perinephric area (Figure 1); (ii) Free Fluid: large fluid collection in perinephric area extending along the ureter in the retroperitoneum (Figure 2); (iii) Urinoma: encapsulated fluid collection surrounded by a fibrous capsule (Figure 3).

Overall, 66 consecutive individuals evaluated at a single centre and treated between September 2014 and October 2020 were considered for final analysis. 

Data collection followed the principles outlined in the Declaration of Helsinki. All patients signed an informed consent agreeing to share their own anonymous information for future studies. The study was approved by the Foundation IRCCS Ca’ Granda–Maggiore Policlinico Hospital Ethical Committee (Prot. 25508).

### 2.2. Statistical Analyses

The distribution of data was tested with the Shapiro–Wilk test. Descriptive statistics of categorical variables focused on frequencies and proportions. Medians and interquartile ranges (IQR) were reported for continuously coded variables. First, interobserver agreement for radiological score was evaluated with the Intraclass Correlation Coefficient (ICC) using a two-way mixed model and absolute agreement type. Cohen’s Kappa analyses was also performed to assess interrater reliability among pairs of evaluators.

Second, the Mann–Whitney U test and Chi Square test were used to assess potential differences in terms of clinical, laboratory and radiographic parameters among the whole cohort according to sUUTR severity (local spread vs. free fluid/urinoma). 

Lastly, univariate and multivariate logistic regression analysis were used to test the association between clinical, radiological and serum variables with the absence of contrast medium extravasation during intraoperative pyelography.

Statistical tests were performed using SPSS v.26 (IBM Corp., Armonk, NY, USA). All tests were two sided, with a significance level set at 0.05.

## 3. Results

Table 1 depicts clinical characteristics of the study cohort. 

Overall, main reason for ED admission was renal colic (66.7%), followed by unspecific abdominal pain (18.2%) and fever (10.6%). Median stone diameter was 5 (4–7.7) mm and stones were located in the proximal, mid and lower ureter in 16 (24.2%), 10 (15.2%) and 40 (60.6%) cases, respectively. sUUTR was treated with ureteral catheter placement in 61 (92.4%) cases. Intraoperative extravasation of contrast medium during pyelography was confirmed in only 38 (57.6%) patients. Only two (3.0%) patients developed postoperative Clavien–Dindo grade III complications (Table 1).

Table 2 shows the interobserver agreement for sUUTR classification among radiologists and the urologist.

Overall, the ICC (95% CI) for sUUTR scoring among the three evaluators was high (0.92; 0.88–0.94) showing excellent interrater reliability. Similarly, the ICC among radiologists [0.88 (0.81–0.93)] and between the urologist with Radiologist 1 [0.87 (0.79–0.92)] and Radiologist 2 [0.86 (0.78–0.92)] were good. Similarly, Cohen’s Kappa analysis confirmed that interrater reliability among evaluators was good (all k > 0.7). Supplementary File S1 reports examples of images with different scores according to the reader.

sUUTR scoring from Radiologist 1 was selected as reference standard. Local spread, free fluid and urinoma were found in 24 (36.4%), 39 (59.1%) and 3 (4.5%) patients, respectively.

Table 3 depicts descriptive statistics of the study cohort according to sUUTR severity.

A higher rate of CCI ≥ 1 was found in patients with free fluid/urinoma than those with local spread (40.5% vs. 16.7%, *p* = 0.04). No differences in clinical and laboratory parameters were seen according to sUUTR severity. Patients with free fluid/urinoma more frequently showed intraoperative extravasation of contrast medium during pyelography than those with local spread (71.4% vs. 33.3%, *p* < 0.01). No differences in clinical or laboratory parameters were seen between patients with and without urine extravasation during surgery (Supplementary Table S1).

Table 4 depicts univariable (UVA) and multivariable (MVA) logistic regression models testing the associations between clinical variables and absence of urine extravasation during intraoperative pyelography.

At UVA, only local spread (OR 5.1; 95% CI 1.54–10.17) was independently associated with absence of urine extravasation. MVA logistic regression analysis confirmed that local spread (OR 4.5; 95% CI 1.35–8.14) was the only independent predictor of absence of intraoperative urine extravasation, after accounting for stone size and degree of hydronephrosis (Table 4).

## 4. Discussion

Although sUUTR has been described as a rare entity, when rupture of the excretory system occurs, prompt identification and characterisation of this condition are paramount in order to allow an equally rapid and appropriate management of the disease.

Of note, sUUTR diagnosis represents a challenge for most urologists due to more than one reason. Firstly, literature on the topic is relatively scarce and mostly constituted case reports and few reviews. Moreover, European Association of Urology (EAU) guidelines do not report sUUTR as a complication of urolithiasis [19], despite it being widely described as its most frequent cause [2,6].

In addition to this, in cases of sUUTR due to urinary stones, its clinical presentation varies to a great extent. According to our study, reason of admission at the emergency department was a renal colic in 66.7% of cases. Of note, 18.2% of patients presented with non-specific abdominal pain and 10.6% presented only fever at admission, which poses a diagnostic challenge for urolithiasis and even more so for upper urinary tract rupture.

Noticeably, symptoms at presentation for each patient not only differ in type but also in severity. In our study, no clinical nor laboratory parameter was identified as an independent predictor of leakage severity. Nevertheless, 40.5% of free fluid/urinoma patients had a CCI ≥ 1 with respect to 16.7% of local spread patients. Hence, the presence of comorbidities seems to predispose to a more severe urine leakage, whereas clinical and laboratory parameters do not.

Of note, according to our results, 63.6% of patients with sUUTR presented with grade I–II hydronephrosis, whereas only 36.4% had grade III–IV hydronephrosis. A reasonable explanation of this phenomenon is that because higher degrees of hydronephrosis usually develop slowly over time, the forniceal tissue is able to adapt to the newly imparted tensile strength, without breaking. Moreover, when rupture occurs, urine leaks out of the pelvis in the perirenal space, halting the evolution of hydronephrosis.

In our cohort, median stone diameter was 5 mm and 24.2% of stones were located in the proximal portion of the ureter, 15.2% in the mid portion and 60.6% in the lower ureter. This is in line with previously reported findings [2,6,20] and, as previously stated [2], may be due to a selection bias. Although small stones are likely to be expelled, when they obstruct, they most frequently do so at the level of the vesicoureteral junction. Additionally, small stones are likely to quickly pass through the upper excretory system and stop abruptly at the vesicoureteral junction. This causes a rapid surge of pressure, which occurs acutely, leading to sUUTR.

In conclusion, according to our findings, clinical presentation, laboratory findings and stone characteristics are not reliable predictors of sUUTR severity. This lack of association means that, in clinical practice, we cannot rely only on clinical and laboratory parameters to assume sUUTR severity, but imaging is always needed.

In order to aid urologist to better identify, describe and treat upper urinary tract rupture, Spinelli et al. [6] proposed a novel radiological classification system describing the extent of urine extravasation after disruption of the upper excretory system on the basis of CECT findings.

CECT with delayed acquisitions, where images are obtained 15–20 min after contrast medium administration, is used to identify and classify sUUTR. CECT is considered positive for urine leakage when attenuation in the perinephric or retroperitoneal spaces increases from 0–20 Hounsfield Units (HU) to 200 HU after injection of contrast [7].

The classification system proposed by Spinelli et al. [6], and further assessed in the present study, can be used to describe univocally and in a standardised fashion urine leakage from the excretory system into the perinephric space. It recognises three grades of leakage: local spread, free fluid and urinoma. In local spread, fluid collects and remains confined in the perirenal fat (i.e., adipose capsule of the kidney). When urine percolates further in the retroperitoneum, the leakage is defined as free fluid, whereas when this collection of fluid increases its dimensions but with a fibrous capsule is called urinoma.

The aim of our study was to validate the proposed classification by testing interobserver agreement for this novel sUUTR classification among two radiologists and one urologist. We demonstrated that the ICC for sUUTR scoring among the three evaluators was high (0.92; 0.88–0.94). Similarly, the ICC among radiologists [0.88 (0.81–0.93)] and between the urologist and Radiologist 1 [0.87 (0.79–0.92)] and Radiologist 2 [0.86 (0.78–0.92)] were good. This shows an excellent interobserver agreement among different specialists, making this novel classification system user friendly and easily applicable to the everyday clinical practice.

According to the most experienced radiologist, local spread, free fluid and urinoma were found in 24 (36.4%), 39 (59.1%) and 3 (4.5%) patients, respectively. Of note, urine extravasation was only confirmed in 57.6% of patients during the pyelogram performed during surgery. Interestingly, contrast leakage at nephrostomy tube or ureteral catheter placement occurred more frequently with higher degrees of extravasation. As a matter of fact, 71.4% of patients with free fluid/urinoma showed intraoperative extravasation of contrast medium, compared to only 33.3% of those with local spread. At MVA, local spread was the only independent predictor of absence of extravasation during surgery.

This notion carries important clinical implications—the proposed classification system may guide urologists in the choice of sUUTR treatment. In fact, because of the lack of standardised management of sUUTR due to ureteral stones, the most commonly adopted treatment modality is urinary decompression, irrespective of the severity of the rupture. This might lead to unnecessary anaesthesia, radiation exposure and patient discomfort, particularly in local spread cases, where intraoperative urine extravasation is less frequently found. Therefore, we believe that our initial results are relevant from a clinical standpoint. In fact, patients with local spread may be treated conservatively, without requiring urinary derivation aimed at extravasation control. As a matter of fact, ureteroscopy or active surveillance might be feasible options in these cases. In light of this, since October 2020, patients with local spread have been treated conservatively at our institution (*n* = 4) and we are collecting prospective data to describe our experience in future studies.

Our study is not devoid of limitations. It was designed as a retrospective, non-randomised study, with the intrinsic limitations of its nature. Moreover, it describes the experience of a single hospital, with a relatively small cohort that might have influenced the lack of association between predictors and sUUTR severity; therefore, larger studies across different centres are needed in order to confirm our findings.

## 5. Conclusions

The recent classification score of sUUTR had good inter/intra-reader reliability among radiologists and urologists. Clinical presentation, degree of hydronephrosis and stone characteristics are not reliable predictors of sUUTR severity, whereas the presence of comorbidities predisposes to a more severe urine extravasation. In approximately 40% of cases, urine extravasation was not confirmed during retrograde pyelography. Patients with local spread had five times more chance of absence of urine extravasation than those with free fluid/urinoma, thus suggesting that ureteroscopy or active surveillance might be feasible options in these cases.

## Figures and Tables

**Table 1:** Demographic Characteristics of the Study Cohort (No. = 66).

Age (Years)	
Median (IQR)	58.0 (41–66)
Range	18–88
Gender (No. (%))	
Male	41 (62.1)
Female	25 (37.9)
BMI (kg/m2)	
Median (IQR)	25.6 (22.8–28.5)
Range	19.8–35.7
CCI (value)	
Median (IQR)	0.0 (0.0–1.0)
Mean (SD)	0.7 (1.0)
Range	0–5
CCI ≥ 1 (No. (%))	21 (31.8)
Reason for presentation (No. (%))	
Renal colic	44 (66.7)
Fever	7 (10.6)
Unspecific abdominal pain	12 (18.2)
Other	3 (4.5)
Stone size (mm)	
Median (IQR)	5.0 (4.0–7.7)
Range	2.0–12.0
Stone location (No. (%))	
Proximal ureter	16 (24.2)
Mid ureter	10 (15.2)
Lower ureter	40 (60.6)
Degree of hydronephrosis (No. (%))	
I–II	42 (63.6)
III–IV	24 (36.4)
Max body temperature (Celsius degree)	
Median (IQR)	36.0 (36–37)
Range	36.0–39.2
White blood cells count (×10^3^/mmc)	
Median (IQR)	10.8 (7.4–13.3)
Range	2.2–24.3
C-reactive protein (mg/dL)	
Median (IQR)	0.9 (0.1–7.6)
Range	0.1–31.6
Serum creatinine (mg/dL)	
Median (IQR)	1.2 (0.9–1.5)
Range	0.6–5.0
Time from ED access to surgery (hours)	
Median (IQR)	13 (9–21)
Range	2.0–109
Type of treatment (No. (%))	
Ureteral catheter	61 (92.4)
Nephrostomy tube	5 (7.6)
Intraoperative urine extravasation (No. (%))	38 (57.6)
Postoperative complications (No. (%))	
None	58 (87.9)
Clavien–Dindo I–II	6 (9.1)
Clavien–Dindo III	2 (3.0)
Bladder catheterisation time (days)	
Median (IQR)	6.5 (3.0–8.0)
Range	1.0–26.0
Hospital stay (days)	
Median (IQR)	3.0 (2.0–6.0)
Range	1.0–15.0

Keys: BMI = body mass index; CCI = Charlson Comorbidity Index; ED = Emergency Department.

**Table 2:** Intraclass Correlation Coefficient Test for Interobserver Agreement.

sUUTR Score	Radiologist 1	Radiologist 2	Urologist
Local spread	24 (36.4%)	27 (40.9%)	26 (39.4%)
Free Fluid	39 (59.1%)	36 (54.5%)	35 (53.0%)
Urinoma	3 (4.5%)	3 (4.5%)	5 (7.6%)
Cohen’s Kappa		0.74 *	0.72 *; 0,70 §
ICC (95% CI)		0.88 (0.81–0.93) *	0.87 (0.79–0.92) *; 0.86 (0.78–0.92) §

Keys: ICC = Intraclass Correlation Coefficient; * versus Radiologist 1 according to Cohen’s Kappa analysis, § versus Radiologist 2 according to Cohen’s Kappa analysis.

**Table 3:** Descriptive Statistics of the Study Cohort According to sUUTR Severity (No. = 66).

	Local Spread	Free Fluid/Urinomas	*p* Value *
No. of patients (No. (%))	24 (36.4)	42 (63.6)	
Age (years)			0.7
Median (IQR)	57.0 (41–64)	58.0 (41–66)	
Range	23–82	18–88	
Gender (No. (%))			0.1
Male	12 (50.0)	29 (69.0)	
Female	12 (50.0)	13 (31.0)	
BMI (kg/m2)			0.8
Median (IQR)	25.4 (22.9–28.5)	25.5 (21.9–28.5)	
Range	19.8–34.3	19.9–35.7	
CCI ≥ 1 (No. (%))	4 (16.7)	17 (40.5)	0.04
Reason for presentation (No. (%))			0.6
Renal colic	17 (70.8)	27 (64.3)	
Fever	3 (12.5)	4 (9.5)	
Unspecific abdominal pain	4 (16.7)	8 (19.0)	
Other	0 (0.0)	3 (7.1)	
Stone size (mm)			0.6
Median (IQR)	5.0 (4.0–7.0)	5.0 (4.0–8.0)	
Range	2.0–12.0	2.0–11.0	
Stone location (No. (%))			0.7
Proximal ureter	6 (25.0)	10 (23.9)	
Mid ureter	4 (16.6)	6 (14.2)	
Lower ureter	14 (58.4)	26 (61.9)	
Degree of hydronephrosis (No. (%))			0.4
I–II	14 (58.3)	28 (66.6)	
III–IV	10 (41.7)	14 (33.4)	
Max body temperature (Celsius degree)			0.7
Median (IQR)	36.0 (36–37)	36.0 (36–37)	
Range	36.0–39.2	36.0–38.0	
White blood cells count (×10^3^/mmc)			0.9
Median (IQR)	11.0 (7.3–13.3)	10.2 (8.2–13.1)	
Range	2.2–17.7	3.2–24.3	
C-reactive protein (mg/dL)			0.4
Median (IQR)	0.6 (0.1–4.6)	1.3 (0.1–7.8)	
Range	0.1–31.6	0.1–29.4	
Serum creatinine (mg/dL)			0.6
Median (IQR)	1.2 (0.8–1.6)	1.3 (0.9–1.6)	
Range	0.6–2.1	0.6–5.0	
Time from ED access to surgery (hours)			0.3
Median (IQR)	14 (10–23)	12.5 (8.2–19.7)	
Range	2.0–109	4.0–48	
Type of treatment (No. (%))			0.1
Ureteral catheter	24 (100)	37 (88.1)	
Nephrostomy tube	0 (0.0)	5 (11.9)	
Urine extravasation (No. (%))	8 (33.3)	30 (71.4)	0.003
Postoperative complications (No. (%))			0.8
None—Clavien–Dindo I	22 (91.7)	38 (90.5)	
Clavien–Dindo II–III	2 (8.3)	4 (9.5)	
Bladder catheterisation time (days)			0.6
Median (IQR)	7.0 (2.0–9.0)	6.0 (3.0–8.0)	
Range	1.0–26.0	1.0–13.0	
Hospital stay (days)			0.7
Median (IQR)	3.0 (2.0–6.0)	3.0 (2.0–5.5)	
Range	1.0–15.0	1.0–15.0	

Keys: BMI = body mass index; CCI = Charlson Comorbidity Index; ED = Emergency Department; * *p* value according to the Mann–Whitney test for continuous data and the Chi Square Test for categorical variables, as indicated.

**Table 4:** Logistic regression models predicting absence of urine extravasation at pyelography.

UVA Model			MVA Model	
	OR, (95% CI)	*p*-Value	OR, (95% CI)	*p*-Value
Age	1.1, (0.96–1.03)	0.9		
BMI	1.0, (0.90–1.15)	0.8		
Female gender	1.1, (0.46–3.03)	0.9		
CCI ≥ 1	0.4, (0.13–1.27)	0.1		
Stone size	1.1, (0.85–1.34)	0.5	1.2, (0.86–4.32)	0.4
Grade I–II	1.2, (0.85–1.44)	0.4	1.1, (0.86–4.34)	0.6
Vs. grade III–IV hydronephrosis				
Max body temperature	0.8, (0.41–1.41)	0.4		
White blood cells count	0.9, (0.87–1.11)	0.1		
C-reactive protein	0.9, (0.90–1.05)	0.5		
Serum creatinine	1.1, (0.52–2.27)	0.8		
Time to ED access	1.1, (0.97–1.03)	0.7		
to surgery				
Local spread vs.	5.1, (1.54–10.17)	<0.01	4.5, (1.35–8.14)	0.01
Free fluid/Urinoma				

Keys: UVA = Univariate model; MVA = Multivariate model, BMI = body mass index; CCI = Charlson Comorbidity Index; ED = Emergency Department; OR = odds ratio; CI = Confidence interval.

## Data Availability

Data are available on request due to restrictions, e.g., privacy or ethical reasons.

## Supplementary Materials

The following are available online at https://www.mdpi.com/article/10.3390/diagnostics11091568/s1, Table S1: Descriptive statistics of the study cohort according to the presence of urine extravasation during surgery (No. = 66).
