# Supplementary material for: Spontaneous Upper Urinary Tract Rupture Caused by Ureteric Stones: Clinical Characteristics and Validation of a Radiological Classification System
Source: Diagnostics (Basel). 2021 Aug 29;11(9):1568. doi: 10.3390/diagnostics11091568 (PMC8471042; doi:10.3390/diagnostics11091568)
Supplement: Supplementary file 1 [file diagnostics-11-01568-s001.zip › sUUTR 2.0 - Supplementary File 1 - 18 8 21.pdf]

Examples of images with different score according to the reader

1.

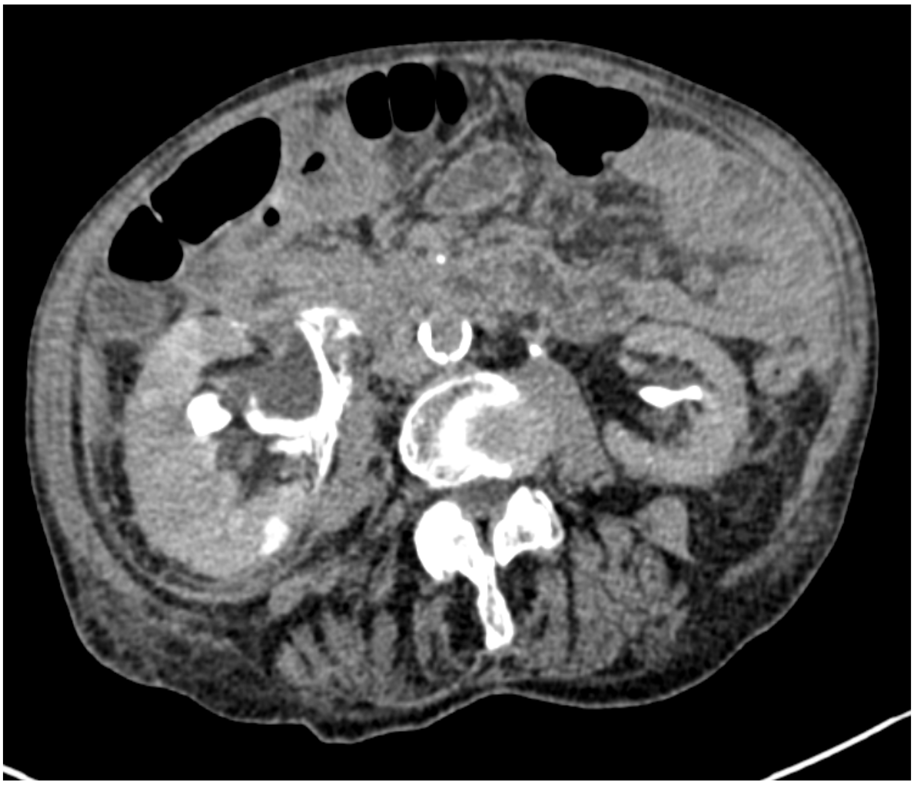

Radiologist 1: free fluid

Radiologist 2: free fluid

Urologist: local spread

2.

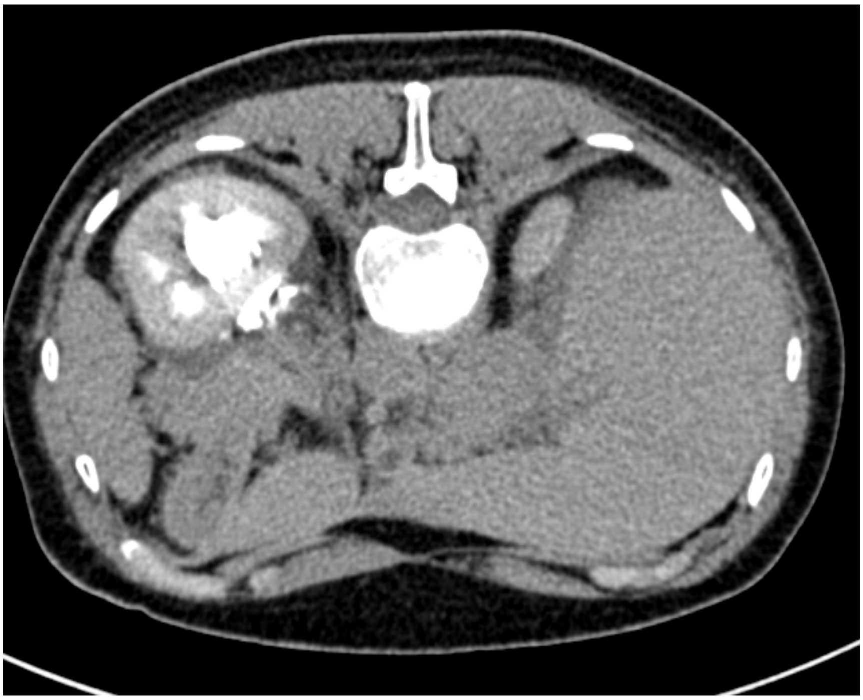

Radiologist 1: local spread

Radiologist 2: free fluid

Urologist: free fluid

3.

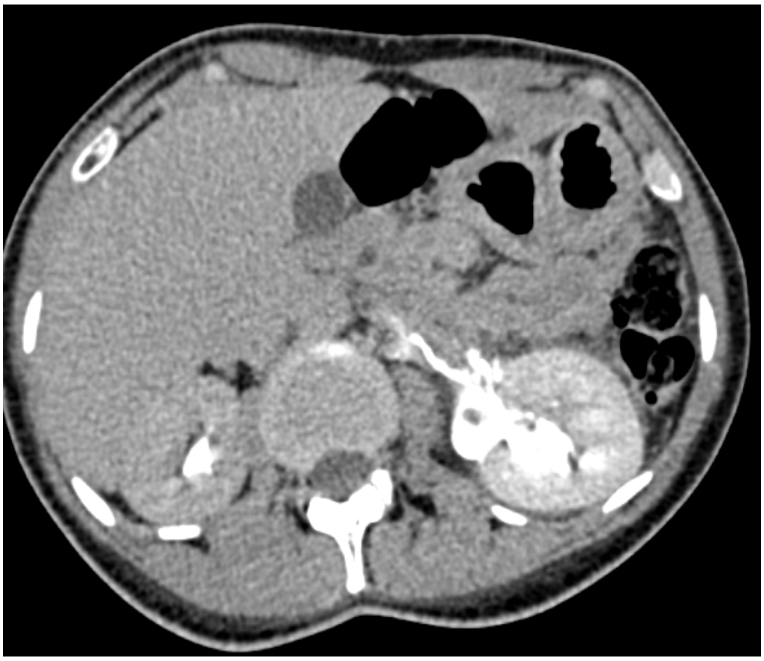

Radiologist 1: free fluid

Radiologist 2: local spread

Urologist: local spread

4.

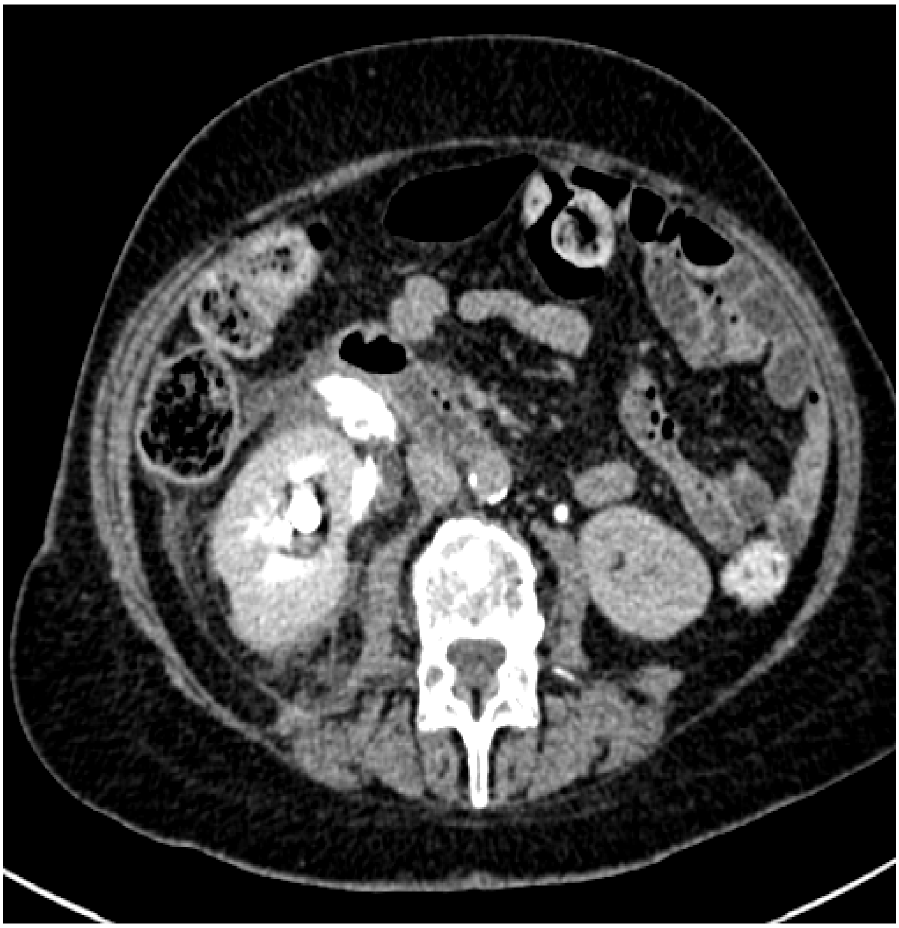

Radiologist 1: free fluid

Radiologist 2: urinoma

Urologist: free fluid

5.

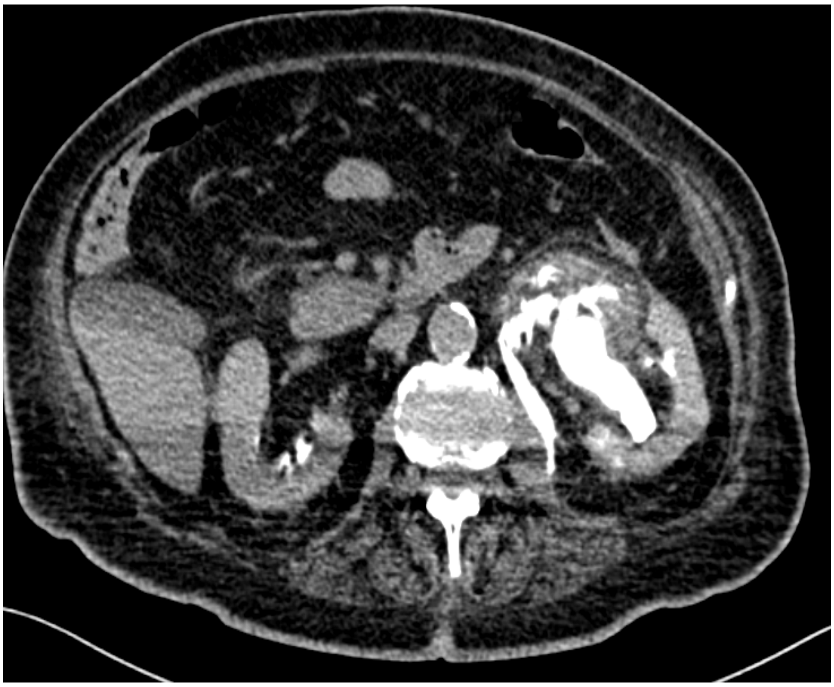

Radiologist 1: free fluid

Radiologist 2: local spread

Urologist: local spread

6.

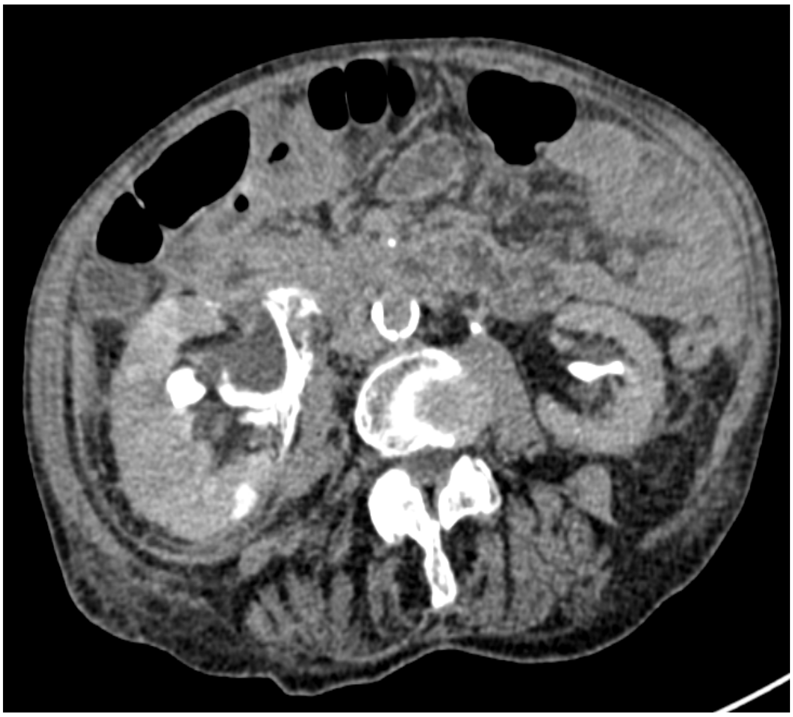

Radiologist 1: local spread

Radiologist 2: free fluid

Urologist: local spread

7.

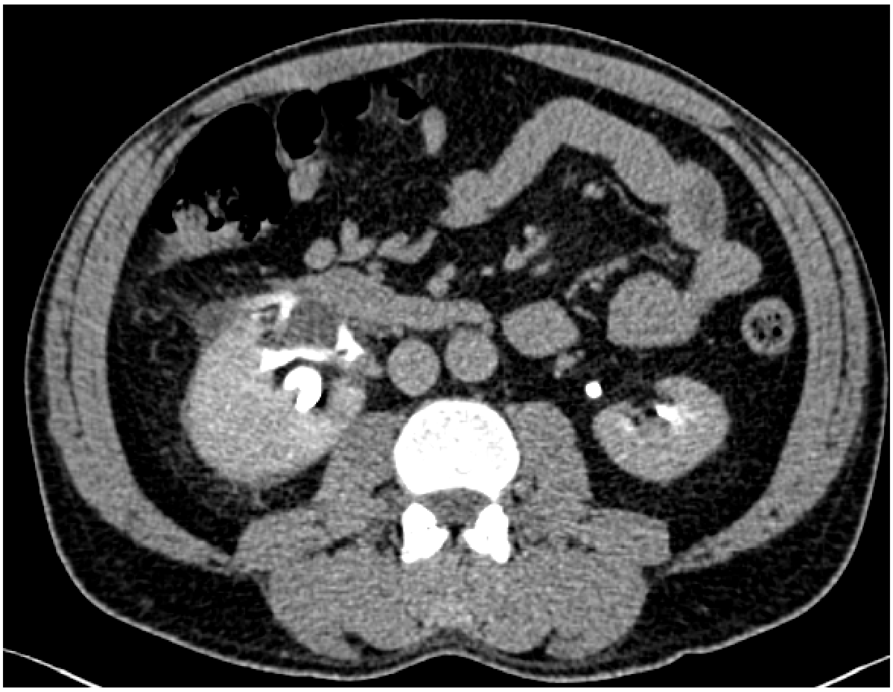

Radiologist 1: local spread

Radiologist 2: local spread

Urologist: free fluid

8.

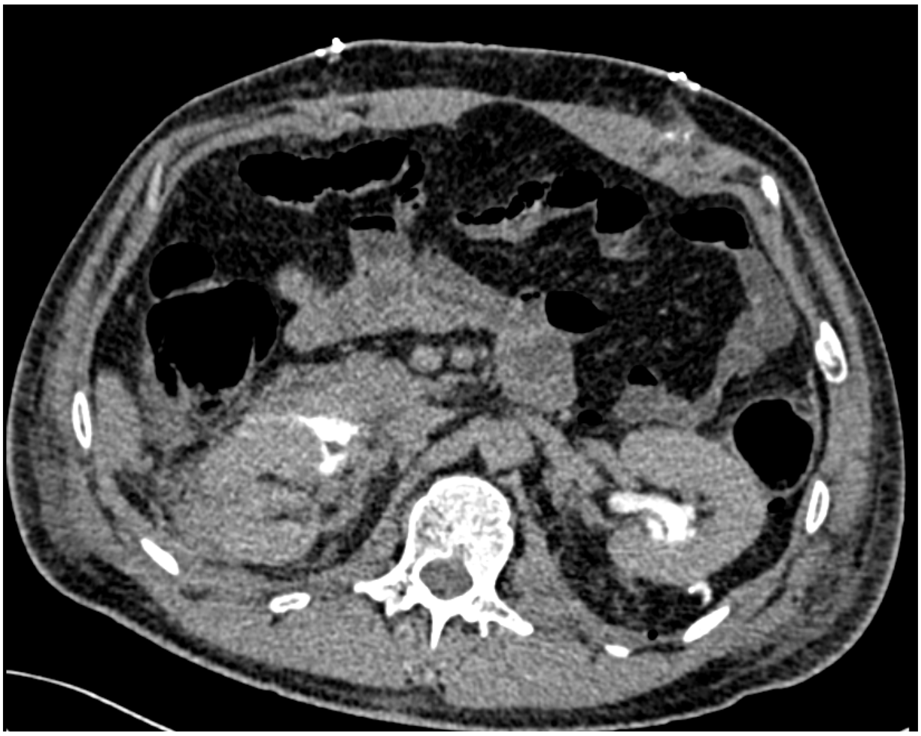

Radiologist 1: free fluid

Radiologist 2: local spread

Urologist: free fluid
