# Supplementary material for: Spontaneous Upper Urinary Tract Rupture Caused by Ureteric Stones: Clinical Characteristics and Validation of a Radiological Classification System
Source: Diagnostics (Basel). 2021 Aug 29;11(9):1568. doi: 10.3390/diagnostics11091568 (PMC8471042; doi:10.3390/diagnostics11091568)
Supplement: Supplementary file 1 [file diagnostics-11-01568-s001.zip › sUUTR 2.0 - Supplementary Table 1 - 3 5 21.pdf]

Supplementary Table S1: Descriptive statistics of the study cohort according to the presence of urine extravasation during surgery (No. = 66)

	+Urine extravasation	-Urine extravasation	p value*
No. of patients [No. (%)]	38 (57.6)	28 (42.4)	
Age (years)			0.8
Median (IQR)	58.0 (43-65)	57.0 (39-65)	
Range	28 - 83	18 – 88	
Gender [No. (%)]			0.8
Male	24 (63.2)	17 (60.7)	
Female	14 (36.8)	11 (39.3)	
BMI (kg/m^2)			0.9
Median (IQR)	25.6 (23.0-28.9)	25.5 (21.8-29.5)	
Range	19.8 – 34.6	21.0 – 35.7	
CCI ≥1 [No. (%)]	15 (39.5)	6 (21.4)	0.1
Reason for presentation [No. (%)]			0.4
Renal colic	24 (63.2)	20 (71.4)	
Fever	6 (15.8)	1 (3.6)	
Unspecific abdominal pain	7 (18.4)	5 (17.9)	
Other	1 (2.6)	2 (7.1)	
Stone size (mm)			0.8
Median (IQR)	5.0 (4.0-7.0)	5.0 (4.0-8.0)	
Range	2.0 – 10.0	2.0 – 12.0	
Stone location [No. (%)]			0.6
Proximal ureter	9 (23.6)	7 (25.0)	
Mid ureter	7 (18.4)	3 (10.7)	
Lower ureter	22 (58.0)	18 (64.3)	
Degree of hydronephrosis [No. (%)]			0.9
I - II	24 (63.1)	18 (64.2)	
III – IV	14 (36.9)	10 (35.8)	
Max body temperature (Celsius degree)			0.7
Median (IQR)	36.0 (36-37)	36.0 (36-36)	
Range	36.0 – 39.0	36.0 – 39.2	
White blood cells count (x10^3/mmc)			0.2
Median (IQR)	9.5 (7.4-12.3)	11.2 (7.5-14.5)	
Range	2.2 – 17.2	2.2 – 24.3	
C-reactive protein (mg/dL)			0.9
Median (IQR)	0.7 (0.1-8.0)	1.8 (0.2-7.8)	
Range	0.1 – 31.6	0.1 – 20.3	
Serum creatinine (mg/dL)			0.9
Median (IQR)	1.2 (0.9-1.6)	1.3 (0.9-1.6)	
Range	0.6 – 3.4	0.6 – 5.0	
Time to ED access to surgery (hours)			0.9
Median (IQR)	14 (9-22)	12.5 (9.0-21.0)	
Range	2.0 – 48	4.0 - 109	
Type of treatment [No. (%)]			0.4
Ureteral catheter	35 (92.1)	26 (92.8)	
Nephrostomy tube	3 (7.9)	2 (7.2)	
Type of sUUTR			<0.01
Local Spread	8 (21.1)	16 (57.1)	
Free Fluid/Urinomas	30 (78.9)	12 (42.9)	
Postoperative complications [No. (%)]			0.2
None - Clavien Dindo I	36 (94.7)	24 (85.7)	
Clavien Dindo II - III	2 (5.3)	4 (14.3)	
Bladder catheterization time (days)			0.8
Median (IQR)	6.0 (4.0-8.0)	7.0 (2.0-8.0)	
Range	1.0 – 13.0	1.0 – 26.0	

Hospital stay (days)			0.2
Median (IQR)	3.0 (2.0-5.0)	3.0 (2.0-8.0)	
Range	1.0 – 15.0	1.0 – 15.0	

Keys: BMI = body mass index; CCI = Charlson Comorbidity Index; ED = Emergency Department; sUUTr = Spontaneous upper urinary tract rupture.

*P value according to the Mann-Whitney test for continuous data and the Chi Square Test for categorical variables, as indicated
